# Word association task responses prime associations in subsequent trials

**DOI:** 10.1177/17470218241239321

**Published:** 2024-03-24

**Authors:** David Playfoot, Ondrej Burysek

**Affiliations:** School of Psychology, Swansea University, Swansea, UK

**Keywords:** Word association, priming, semantic networks

## Abstract

The word association task has been used extensively in psychological and linguistic research as a way of measuring connections between words in the mental lexicon. Interpretation of word association data has assumed that responses represent the strongest association between cue word and response, but there is evidence that participant behaviour can be affected by task instructions and design. This study investigated whether word association responses can be primed by the participants’ own response to the preceding cue—that is, whether the order in which cues are presented alters the responses that are generated. Results showed that the proportion of participants who provide a particular association (e.g., acid—RAIN) is greater when their response to the previous cue in the list is also associated with rain (e.g., parasol—UMBRELLA). The same is not true when the two cues are presented non-consecutively. Word association tasks should be administered such that the order in which cues are presented is random for every participant so as to avoid unintentional contamination of associative strength data.

The word association task was first developed in 1879 to assess individual differences in IQ (Galton, 1879). The *discrete* version of the task, as used in the current studies, is generally administered by presenting a cue stimulus (usually a word), orally or visually, and the respondent is asked to say or write the first word he or she associates with the cue. Since its development, the word association task has been used to uncover unconscious conflicts or drives that are otherwise inaccessible (Jung, 1910; Vezzoli et al., 2007), to test for “insanity” (Kent & Rosanoff, 1910) and to identify prejudices that participants would otherwise try to hide (Rojas-Rivas et al., 2022). Perhaps the most extensive use of the word association task is in psycholinguistics where responses in word association tasks have informed what we know about the structure and organisation of semantic memory and the mental lexicon (De Deyne & Storms, 2008). In all of these contexts, the underlying assumption is that the word that the participant produces is the strongest related concept in their mind and that it is produced without much interference or metacognitive control being exerted. This is an assumption that has not been empirically tested, with a few notable exceptions, and the studies described in the current paper aim to address this gap in the literature.

The word association task is based on the premise that the words and concepts that an individual knows are stored in a network of interconnected nodes. These *semantic network models* (e.g., Collins & Loftus, 1975; Steyvers & Tenenbaum, 2005) posit that each word node is connected to one or more other word nodes with similar meaning. The links between word nodes vary in strength according to the experience of the individual—words that frequently co-occur, for example, have stronger links than pairs of words that are only tangentially related. When a word is encountered (e.g., when it is presented as a cue in a word association task), its node is *activated* in the lexical network, and some activation spreads to other connected nodes as a function of the strength of the link. As the greatest amount of activation should be passed from the activated cue along the strongest intra-lexical link, the “first word that comes to mind” in a word association task ought to reflect the most strongly associated concept (De Deyne et al., 2019; Playfoot et al., 2018).

Although the precise structure and organisation of the lexical network varies from person to person based on their own idiosyncratic language experience, there are commonalities across participant samples which have been used to create word association norms lists (e.g., De Deyne et al., 2019; Kiss et al., 1973; Nelson et al., 2004). These norms lists reflect both the variety of associative responses that are offered by participants to a given cue and *associative strength* of a response, defined as the proportion of participants who provided the same response to the same cue. It is common for semantic network diagrams or models to be created on the basis of word association norms lists (e.g., De Deyne & Storms, 2008; Steyvers & Tenenbaum, 2005) and often the associative strength data are used as a metric by which to determine which nodes are included or excluded, or how strong the links between the nodes are assumed to be. Importantly, if the assumption that word association responses reflect the strongest intra-lexical link between two nodes is incorrect then these semantic network models are incorrect as well. There are two ways that the critical underlying assumption of word association tasks could be incorrect—either (1) the response could reflect a word *other than* the first to come to mind or (2) the response that is elicited could be *influenced* by something other than the presented cue on that trial.

The first of these possibilities has already been examined by Playfoot et al. (2018). They conducted two experiments in which participants were asked to complete a standard word association task plus another task using the same cues but different instructions. In one case, the “creative” association task, participants had to generate responses which were legitimately related to the cue but which they thought would not be offered by other participants in the experiment; in the other case, participants had to provide word associations under time pressure so as to preclude them from offering a response other than the first word that came to mind. Responses in the creative word association task took significantly more time to generate than responses in the standard word association task, which was argued to indicate that the creative word association task required time for activation to spread to nodes that were not as strongly connected to the cue in the lexical network. When asked to respond under time pressure, the likelihood of generating the response with the highest associative strength was increased (as a proportion of total responses generated). In other words, participants found it hard to generate a response *other than* the first word that came to mind when additional constraints were placed on the task.

The second possible downfall of the assumptions underlying the word association task is that the responses that are generated are influenced by factors outside of the specific cue that is presented. Some of these factors will be outside of the control of the researcher (e.g., news stories or popular culture factors that temporarily alter the dominant sense of a cue word). However, there are also potential factors about the experimental design that could influence the associations that participants generate. These issues, too, are based on the spreading of activation in the lexical network. In priming tasks, the presentation of a stimulus (even briefly) can influence the processing of a subsequent target stimulus presumably because spreading activation has prepared the target for retrieval to some extent. Semantic priming is among the most reliably observed phenomena in cognitive psychology (Maxfield, 1997). For example, in the lexical decision task, it can influence the response time and/or accuracy of differentiating a word from a non-word (Perea & Rosa, 2002). This has been observed in bilingual populations across different languages (Wen & van Heuven, 2017), or even in Alzheimer’s patients (Nebes et al., 1989). Furthermore, a large body of evidence shows that priming can occur even when the prime is not perceived consciously (Bodner & Masson, 2003; Van den Bussche et al., 2009; Wen & van Heuven, 2017).

In the context of word association tasks, studies have successfully primed both more and less frequent meanings of semantically ambiguous cue (e.g., bank; Betts et al., 2018; Gilbert et al., 2018; Rodd et al., 2016). For instance, in Rodd et al.’s (2013) paradigm, the participants were exposed to spoken sentences that disambiguated homonymous cues in line with their less dominant meaning (e.g., “the seal came up onto the bank of the river”). After each sentence, a visually presented word that either was (e.g., shore) or was not (e.g., tragic) related in meaning was presented, and participants were required to make semantic relatedness decisions. Following this phase of the experiment participants were given a filler task and in the critical final phase participants were asked to complete a word association task in which the homonyms were used as cues. Rodd et al. (2013) found that participants who had heard the disambiguating sentences were significantly more likely to offer a response related to the less dominant meaning of the homonyms than participants who had not heard the sentences. Other studies also show that priming effects can elicit responses related to a certain aspect of the target cue even when the cue is not a homonym. For example, when the cue BEACH is primed for being warm, it is more likely to elicit response like SUN, than responses like SAND (Curtis et al., 2022). However, in all of the studies mentioned above, the primed response is evoked by, and possibly dependent on, the relatively extensive priming phase that precedes the word association task. Moreover, the priming phase is often followed by tasks that enhance the processing of the cue, e.g., by judging semantic relatedness (Rodd et al., 2013) or rating the cue on pleasantness (Goshen-Gottstein & Kempinsky, 2001; Wang & Yonelinas, 2012; Zeelenberg et al., 1999). Although this ensures attentiveness and sufficient processing of the cues, it prevents us from extrapolating to word association task itself.

In contrast, there is also evidence that participants’ responses can be influenced by the responses that they themselves have already generated (e.g., De Deyne et al., 2019; McEvoy & Nelson, 1982). This response chaining is commonly a consideration in the use of *continuous* word association tasks, in which participants are asked to give multiple responses to a single cue. For example, De Deyne et al. (2019) showed that when participants were asked to generate three associates to a cue, for instance sun, the likelihood of producing “star” as the second response was significantly higher for those who had produced “moon” as the first response. In other words, the association generated by a participant acted as a prime for their next association response.

We argue, therefore, that it is possible that a form of response chaining could occur across multiple trials in discrete word association tasks. Consider a situation in which a participant is asked for an associate for the cue “parasol” on Trial 1 and responds with “umbrella.” On Trial 2, the cue presented is “acid.” Within the lexical network of that participant, it is likely that the node representing “rain” is connected to both umbrella and to acid. Hence, some activation has been passed to the “rain” node from umbrella (the previous response) *and* from the current cue of acid, and the likelihood of producing rain as a response is increased. Traditionally, word association tasks were completed on paper such that all participants responded to the same cues in the same order. If response chaining as outlined above does influence the associations offered by participants, it is possible that existing (and still popular) norms lists are at least in part artefacts of the way in which the task was administered. The experiments in this article aimed to determine whether cross-trial response chaining could be observed. We describe the process in two separate sections. First, we identified potential sequences of cues in which the response chaining could occur and confirmed that the associative strengths between the respective parts of the sequences were as we expected them to be based on existing norms. Second, we compared the word association responses with the cues in these sequences when participants were presented the cues in sequence versus when they were presented separated by at least two intervening trials. We hypothesised that associative strengths would be greater in the sequential list than they would when cues were presented out of sequence.

## Study 1—generating cue-response sequences

### Participants

We recruited 122 participants via Prolific (2022; https://www.prolific.co/). The participants were 20.98 years old, on average, with a standard deviation of 1.72. Seventy-five of the participants identified as female, 44 as male and 3 preferred not to disclose their gender. Participants were all monolingual speakers of English born in the United Kingdom, and none of them had received a diagnosis of dyslexia or other language disorder.

### Materials and design

To begin, we used the English-language “Small World of Words” norms list (De Deyne et al., 2019) to identify the most common responses (which will be referred to as R1 hereafter) to a large number of word association cues (C1 hereafter). We then searched to determine whether those common responses (R1s) were also in the norms list as cues themselves and noted the most common response to those cues as well (we refer to these as R2s). Finally, we searched the database for instances where the R2s had been generated to other cues. These “other” cues were potential candidates to be presented as the second cue (C2) in the sequence. An example of a chain generated in this way is parasol (C1)—umbrella (R1)—rain (R2)—acid (C2). From this initial scoping of the norms list, we edited down to 66 potential sequences. To be retained (1) the C1-R1 associative strength had to be greater than .3, (2) the R1-R2 associative strength had to be greater than .3, and (3) R2 had to be in the top three most common responses to C2, with an associative strength of at least .1. We chose these cut-offs on the basis that for (1) and (2) we wanted a reasonable proportion of participants to give R1 and R2, respectively, in order to have an appropriate sample size to include in the analyses. An associative strength of .3 might appear to be quite a low threshold, but it is important to note that there is a great deal of variability in the frequency with which the most common response occurs from cue to cue. For example, 89.7% of respondents in the Small World of Words study (De Deyne et al., 2019) offered “stop” as an associate for HALT; only 12.1% offered “total” as an associate for ABSOLUTE. Both of these examples were the most common response to their respective cues in the norms list. In Fitzpatrick et al.’s (2015) data, the average associative strength for the most common response was .26. Thus our cut-offs were slightly above the average from that study to give a reasonable expectation that some, but not all, participants would give the expected R1. In relation to the cut-off for (3) we wanted to use cues that elicited the expected response frequently, but that was not such a strong association that we could be limited by ceiling effects later.

The 66 sequences were then split into three equal lists by random number generator. We created three versions of a word association task on the basis of the lists above. In each version, participants were presented with 22 C1s, 22 C2s, and 22 R1s acting as cues, but no more than one constituent of the same sequence so that participants were unlikely to guess the purpose of the task. All three versions also contained the same 34 filler cues drawn from Fitzpatrick et al. (2015). The purpose of including these fillers was twofold. First, it would allow us to check whether the participants given each version of the word association task were responding in broadly similar ways by comparing filler responses across groups. Second, including cues that were not part of potential sequences would further obscure the true purpose of the task. Cues were presented in a random order in each version.

### Procedure

The cues were presented one at a time and were accompanied by a text box into which the participant could type their response. Participants were instructed to write the first word that came to mind and were assured that there were no right or wrong answers. All stimulus presentations were managed using Qualtrics (2020; https://www.qualtrics.com/). It took an average of approximately 10 min for participants to complete the task.

## Results

As a first step in the analysis, responses were cleaned following the procedure used by Fitzpatrick et al. (2015). This meant that spellings for the participants’ responses were corrected in circumstances where what had been typed was (1) not an existing English word and (2) a clear and unambiguous mistake. Non-word responses that were equally close to more than one real word were not amended. Once the spellings had been checked, words were lemmatised according to Level 2 of Bauer and Nations’ (1993) taxonomy. This includes coding words with the same base and different inflections recognised in this level as the same. For example, DEVELOPS and DEVELOPING are both coded as DEVELOP.

Having completed this cleaning of the data, we examined the consistency of responses to the 34 filler cues across the three versions of the task. At least two groups had the same dominant response for 91% of the filler cues and for 18 out of 34 cues the same dominant response was shared in all three groups. Next, we calculated the associative strength for the dominant response given to those 18 cues separately for each group and compared the associative strengths. A one-way ANOVA indicated that there was no significant effect of group on the associative strengths (*F* < 1) and correlations between the associative strengths in pairs of groups revealed *r* values between .88 and .92. This indicates that the three groups were similar in the way that they responded to the common filler cues. As a consequence, we assumed that the word association responses of a particular group for the potential constituents of a cue-response sequence would also be more or less representative of the responses of one of the other groups.

In the next phase of the analysis, we calculated the associative strength of each response that was recorded in relation to the C1, C2, and R2 cues. The purpose of this was to allow us to confirm whether a potential sequence of cue-response pairs would be viable in our target sample. Having calculated the associative strengths, we again applied the selection criteria described in the Materials section to the potential sequences, this time using our own data rather than the estimates from De Deyne et al. (2019). Of the 66 initial chains, 1 was excluded because of an error in the setup of the word association tasks such that one of the constituent words was omitted, 13 were excluded because the associative strength of C1-R1 was < .3, 8 had R1-R2 associative strengths < .3 and 15 had C2-R2 associative strengths < .1. This resulted in 29 remaining sequences, of which 20 were arbitrarily selected for inclusion in Study 2.

## Study 2—examining cross-trial response chaining

The aim of Study 2 was to test the hypothesis that word association responses to a given cue could be affected by the response generated for the preceding cue word. Given that traditional word association tasks have been presented with cues in a fixed order, it is possible that previous norms lists, and semantic network models built on the basis of those norms lists, may have been contaminated by spill-over effects from one trial to the next.

### Participants

According to calculations using GPower 3.1.9.2 (Faul et al., 2007, 2009), 100 participants would be sufficient to detect a small effect at α = .05 and a 1-β power of .8 and we overrecruited in case of incomplete data. One hundred and nineteen participants aged between 18 and 45 (M = 24.24 years; *SD* = 5.7) completed the study. Of these participants 90 were female, 28 were male, and 1 participant preferred not to disclose their sex. Approximately half (59) were recruited from the undergraduate Psychology programme at Swansea University and the remaining 60 were recruited via Prolific (2022; https://www.prolific.co/). Participants recruited from Prolific were given £3 for completing the study; Swansea University students received course credit instead. All participants were fluent English speakers living in the United Kingdom, without diagnosis of dyslexia or any other language difficulty.

### Materials and design

All participants were presented with a total of 85 cues, one at a time, for a word association task. Of these 85 cues, 40 were the C1s and C2s identified in Study 1 and the remaining 45 were filler cues drawn from Fitzpatrick et al. (2015). Participants were randomly allocated to one of the two list orders: randomised or sequenced. In the randomised condition, the order of all cues was initially selected using randomizer.org (Urbaniak & Plous, 2013). In the event that the C1 and C2 from the same sequence were supposed to appear with fewer than two intervening filler cues, irrespective of the order, they were separated by displacing the latter cue to the bottom of the list. If this displacement did not separate the two cues further than two cues away, the latter cue was placed to the top as the first cue in the list instead. In the sequenced list each *pair* of C1 and C2 (as identified in Study 1) was presented to the participants on consecutive trials. Filler cues were added to the list such that one to four cues appeared between each C1 C2 pair so that participants were unlikely to become aware of the pattern of presentation.

### Procedure

Participants completed the experiment online via Qualtrics (2020; https://www.qualtrics.com/). They were instructed to write down the first word that came to mind in response to the cue presented on the screen. An example was provided with a cue that was not used in the experiment. Cues were presented one by one with a textbox for the response located underneath. Participants proceeded to the next trial after they had written a response and clicked “Next.” They were not allowed to proceed without responding, neither were they given any time constraints to respond.

## Results

Preparation of the data followed the same steps as for Study 1—clear and unambiguous spelling errors resulting in non-word responses were corrected, and all responses were lemmatised to the second level of Bauer and Nation (1993). The responses to the filler cues were not analysed any further. One participant was excluded entirely as they had retyped the cues rather than generating associated words, so the analyses reported here are based on 118 participants’ data.

We calculated the proportion of participants who gave the expected response (R1) to the first cues of a sequence (C1s) separately for each list order. Given that C1 was the first of a pair of cues presented in the sequenced condition, and was presented independently of any related cue in the randomised condition, we would expect that a similar proportion of participants would give R1 in both conditions. There was no significant difference between the two conditions, *t*(38) = 0.70, *p* = .491, Cohen’s *d* = 0.22.

The critical comparison for the study was between the likelihood that participants gave the expected R2 to C2 in the two list orders. We also wanted to determine whether the likelihood was greater in those participants who had also given the expected R1 to C1 which we suggest would occur predicted based on hypothetical residual activation within the lexical network from the previous trial. In order to assess this, we ran a generalised multi-level model including random intercepts for cue pair and for participant, entering list order and whether the participant gave the expected R1 as fixed effects. The analysis was conducted in R, using the lme4 package. We first generated a model containing only the random effects to use as a baseline. Following that, we generated a second model including both the fixed and the random effects. The analysis revealed that the second model fit the data significantly better than the baseline model, χ^2^(3) = 55.34, *p* < .001. The effect of the list order (*z* = 1.99, *p* = .046) and the interaction between list order and response to C1 (*z* = 3.01, *p* *=* .003) were both significant. The estimated marginal means are presented in Table 1.

**Table 1.** Mean likelihood of participants giving the expected response to the second cue (R2) in a pair as a function of whether they had also given the expected response to the first cue in a pair, according to cue presentation order. Standard errors are in parentheses.

	Did not give expected R1	Gave expected R1	Total
**Sequenced**	.20 (.04)	.40 (.05)	.30
**Randomised**	.19 (.04)	.24 (.04)	.23
**Total**	.20	.32	

R1 refers to the response to the first cue in a pair. Sequenced = cue pairs were presented on consecutive trials. Randomised = cue pairs were presented with 2 or more intervening cues.

To further examine the interaction, we computed contrasts between pairs of conditions, corrected using Tukey’s method. The likelihood that participants gave R2 in response to C2 was significantly higher in instances where the cue pairs were presented sequentially *and* the participant said the expected R1 than in any other condition (all *p* < .001). There was no significant difference in the proportion of participants who gave the expected R2 across the other three conditions (all *p* > .1).

As a final stage in the analysis, we examined the influence of the associative strength between constituent pairs in the chains (C1-R1, C2-R2 and R1-R2) on the proportions of participants that provided the expected R2. We approached this in two ways. First, within each list order, we calculated the difference between the proportion of participants who said both the expected R1 and the expected R2 and those who gave *only* the expected R2. The resulting difference score (which will be referred to as “R1 influence” hereafter) is an estimate of the overall effect of giving R1 on the likelihood of giving R2 for a given C1 C2 chain. We then correlated R1 influence scores with C1-R1, C2-R2 and R1-R2 associative strengths, respectively. The resulting correlations are reported in Table 2. In each case, the correlation was only significant in the sequenced list condition.

**Table 2.** Correlations between R1 influence and associative strengths of pairs of words within chains, split by list order.

	C1-R1	C2-R2	R1-R2
**Sequenced list**	.29*	.30*	.33*
**Randomised list**	.02	.24	-.01

R1 influence refers to the difference in the proportion of participants who gave the expected R2 depending on whether they also gave the expected R1.

* *p* < .05.

Second, we calculated the difference between the proportion of participants who said both R1 and R2 in the sequenced condition versus the randomised condition. The resulting score (referred to as “list influence” hereafter) was correlated with C1-R1, C2-R2 and R1-R2 associative strengths, respectively. The correlation of list influence with R1-R2 associative strength was significant, *r*(18) = .59, *p* = .007, but neither the correlation with C1-R1 associative strength, *r*(18) = .17, *p* = .467, nor with C2-R2 associative strength, *r*(18) = −.22, *p* = .352, was significant.

In sum, there were significant influences of associative strength only when C1 and C2 were presented on consecutive trials. The R1-R2 associative strength was particularly influential in driving the effect of list order described above.

### Interim discussion

In Study 2, it was found that the likelihood of generating a specific word association response to a cue is higher when this response is primed by the participant’s own answer in the previous trial. In other words, if the response a participant generates to C1 would be likely to elicit the same response as the next cue that is presented, then the associative strength of the C2-R2 relationship is likely to be overestimated. The same is not observed if C1 and C2 are presented on non-consecutive trials. This pattern was observed in spite of the fact that participants in the two list presentation orders showed no significant difference in the associative strength of the C1–R1 pairing (meaning that it is unlikely to be because participants in one condition were more likely to make the “priming” association). Moreover, the two groups showed no significant difference in the likelihood of producing the expected R2 when the expected R1 was not produced. This indicates that the priming effect was present and well-isolated to the initial response stated in the preceding trial. In addition, the correlational analyses indicated that the likelihood of participants generating both R1 and R2 was related to the R1-R2 associative strength. The proportion of participants who gave both R1 and R2 was greater in the sequenced list condition than when cues were randomised, but the magnitude of that increase was greater when R1 and R2 were more closely associated. This further evidences the importance of considering the order in which cues are presented and the potential impact that a response generated by the participant themselves can have on subsequent trials.

Given that Study 2 provided evidence that word associations can be primed by the participant’s own responses, the question arises as to how quickly that priming effect decays, and how many intervening filler cues are necessary for the influence to be extinguished altogether. We had designed Study 2 to ensure that cues that formed a chain were separated by at least two intervening filler cues, but the method by which this was achieved did not allow us to adequately answer this question with the data from Study 2—there was no consistent number of intervening fillers, and in many instances the C2 from a chain was presented before the C1. We therefore conducted a new experiment in order to systematically examine the longevity of the priming effect.

## Study 3—priming effect decay

### Participants

According to calculations using G*Power 3.1.9.2 (Faul et al., 2007, 2009), 114 participants would be sufficient to detect a small effect at α = .05 and a 1-β power of .8. One hundred and eighteen participants aged between 18 and 46 (M = 31.46 years; *SD* = 8.44) were recruited via Prolific (2022; https://www.prolific.co/) for Study 3, slightly over-recruiting in case of participant withdrawal. Participants recruited from Prolific were given £3 for completing the study. All participants were fluent English speakers living in the United Kingdom, without diagnosis of dyslexia or any other language difficulty. Fifty-two of the participants identified as female and 66 as male.

### Materials and procedure

The 20 C1 C2 pairs used in Study 2 were sorted into four groups each containing five chains. For one group of chains, C1 and C2 were presented consecutively. For the other groups of chains, C1 and C2 were separated by one, two, or three intervening filler cues, respectively. The average R1-R2 associative strengths were not significantly different in the four groups of chains, *F*(3, 16) = .01, *p* = .999,—Study 2 had shown that R1-R2 associative strength was significantly correlated with the size of the priming effect in consecutive C1 C2 pairs, so it was important to control this in Study 3. After each C2 was presented, between one and three filler cues (as determined by random number generator) were presented before the next C1. Two filler cues were also presented to start the task, and after the final C2. Therefore, the participants saw 74 filler cues in total, all drawn from Fitzpatrick et al. (2015), plus 40 cues that formed part of chains—there were 114 trials in the experiment overall. All participants saw the same list of cues in the same order (hence number of intervening fillers was varied within participants). The procedure and instructions were the same as in Study 2.

### Results

As in Studies 1 and 2, clear and unambiguous spelling errors resulting in non-word responses were corrected, and all responses were lemmatised to the second level of Bauer and Nation (1993). The responses to the filler cues were not analysed any further. We then ran a generalised multi-level model including random intercepts for cue pair and for participant, entering number of intervening fillers and whether the participant gave the expected R1 as fixed effects. The analysis was conducted in R, using the lme4 package. We first generated a model containing only the random effects to use as a baseline. Following that, we generated a second model including both the fixed and the random effects. The analysis revealed that the second model fit the data significantly better than the baseline model, *χ*^2^ (3) = 29.02, *p* < .001. The effect of the response to C1 (*z* = 4.83, *p* < .001) and the interaction between number of intervening fillers and response to C1 (*z* = −2.56, *p* *=* .011) were both significant. A significantly higher proportion of participants provided the expected R2 response if they had also provided the expected R1 when C1 and C2 were on consecutive trials (*p* = .001) but not if there was even a single intervening filler (one filler *p* = .108; two fillers *p* = .451; three fillers *p* = .997). The proportions are presented in Table 3.

**Table 3.** Proportion of expected responses to the second cue (R2) in a pair as a function of whether the expected response to the first cue in a pair had also been recorded, according to number of intervening fillers between Cue 1 and Cue 2. Standard errors are in parentheses.

	Did not give expected R1	Gave expected R1
**Consecutive**	.19 (.04)	.36 (.06)
**One filler**	.19 (.04)	.30 (.05)
**Two fillers**	.23 (.05)	.32 (.06)
**Three fillers**	.24 (.05)	.27 (.05)

R1 refers to the response to the first cue in a pair.

## General discussion

The current studies have demonstrated that responses in the pen-and-paper word association tasks that have historically informed models of semantic memory and lexical organisation may have been contaminated by the responses that participants had generated to previous cues. Study 2 showed that the likelihood of producing a particular association for a cue is influenced by what was presented (or more accurately, what the participant responded with) on the preceding trial. Study 3 further explored priming effects in word association by manipulating the number of intervening cues presented between C1 and C2 of the same chain. Although there were numerical increases in the proportion of participants who gave the expected R2 after generating the expected R1 in all conditions, we found that even one filler cue was enough to make the priming effect non-significant. Word association tasks are now more likely to be conducted on computers with cues being presented in a random order so cue-order effects are likely to be avoided in future, but the evidence of potential historic confounds is nevertheless important. For example, Steyvers and Tenenbaum’s (2005) model of semantic networks was informed by the word association patterns from pen-and-paper tests (specifically Nelson et al., 2004). The model continues to be widely cited, so while the underpinning word association norms themselves may be out-dated the model is still influential.

The finding that participant responses can be influenced by the experimental context are not surprising given previous literature on priming effects in word association (Curtis et al., 2022; Rodd et al., 2016). They are nonetheless meaningful, because this is the only study, to our knowledge, that has reported a priming effect *without* a priming phase and predicated solely on the participant’s own responses. Our findings can be accounted for by the principle of spreading activation (Collins & Loftus, 1975) in that R1 partially activates the node for R2 in the lexical network so that when C2 is presented R2 is closer to some sort of activation threshold and thus easier to access. The same priming is not observed when C1 and C2 are presented on non-consecutive trials presumably because there has been sufficient time for the initial activation of the R2 node to have been inhibited, or because C2 has preceded C1 in the random order so that R2 is elicited before any activation could have spread at all. In addition, we suspect that the magnitude of the priming effect observed is proportional to how closely the constituent parts of the sequence are linked. Associative strength has previously been shown to predict the magnitude of priming effects in lexical decision (Canas, 1990). In a similar way, we considered that more strongly related C1-R1 pairs would be likely to cause a greater priming effect in generating R2, as would more strongly related R1-R2 pairs. The analysis of the data collected in Study 2 indicated that it is the associative strength of the R1-R2 pairs that is particularly important in driving the priming effect we observed. There are at least two potential accounts for this relationship, both predicated on the assumption that if the activation of R2 is below a criterion level when C2 is presented then no priming effect will be observed. The first is that, for some participants, the connection between R1 and R2 was not sufficiently strong in their own lexicon for much activation to spread from node to node. This would mean that little to no “priming” had ever occurred for those participants and the R2 node remained below the criterion level when C2 appeared onscreen. The second potential account is that the inhibition of the R2 node had returned the activation of R2 to below the criterion level by the time the participant generated their word association response. Assuming that inhibition happens at roughly the same rate across the whole lexical network, R2 nodes that were less strongly activated by generating R1 would take less time to fall below the threshold and would hence be less likely to exhibit priming effects even if they had initially been activated. As associative strength is a measure of group-level behaviour rather than an index of any individual participant, either of these accounts are plausible.

In creating the chains in Study 1, we used cut-offs for inclusion of cue-response pairs based on associative strength from the Small World of Words norms (De Deyne et al., 2019). Although the thresholds for inclusion were informed by knowledge of word association response patterns and have allowed us to address our research questions, they were chosen somewhat intuitively. The question arises as to whether the same patterns would have been observed if a different cut-off had been selected—a higher associative strength threshold would likely have resulted in too few chains being extracted, but it may be that a lower threshold would have provided sufficient chains to explore the issue of priming in greater detail without altering the overall headline findings. This is an empirical question which could be explored in future research. With that said, it is important to remember that the associative strength reported between a cue and a response in a norms list is an estimate of the proportion of times that the response will be generated in a population of participants. This is not the same as a probability of the response being generated by any individual participant. As a result, it would not be possible to estimate the exact influence of the order in which cues have been presented on existing norms lists (or even in this study). Given that participants are only presented with a cue once, we cannot estimate the likelihood that they would have given the critical R2 even in the absence of R1 on the previous trial—the proportion observed in the randomised list provides the best available estimate, but the idiosyncratic nature of word associations and semantic networks means that it will remain *only* an estimate. Nevertheless, the key recommendation of the studies described in this paper is that research using the word association task should *always* present cues in a separate random order for each participant so as to get the purest possible assessment of the strength of relationships between cues and responses.
